# Antifungal Potential of *Artemisia* Essential Oils and Extracts Against Mycotoxigenic Fungi

**DOI:** 10.3390/toxins18100426

**Published:** 2026-10-01

**Authors:** Imene Ghorbal, Sara Redouane-Salah, Safa Smail, Amandine Nachtergael, Mohammed Aufy, Mohamed F. Abdallah

**Affiliations:** 1 Department of Natural and Life Sciences, Faculty of Natural and Life Sciences, Earth and Universe Sciences, University of Biskra, Biskra 07000, Algeria; imen.ghorbal@univ-biskra.dz (I.G.); sara.redouanesalah@univ-biskra.dz (S.R.-S.); safa.smail@univ-biskra.dz (S.S.); 2 Laboratory of Promotion of Agricultural Innovation in Arid Regions, University of Biskra, Biskra 07000, Algeria; 3 Department of Therapeutic Chemistry and Pharmacognosy, Faculty of Medicine, Pharmacy, and Biomedical Sciences, University of Mons, 7000 Mons, Belgium; amandine.nachtergael@umons.ac.be; 4 Department of Pharmaceutical Sciences, Division of Pharmacology and Toxicology, Faculty of Life Sciences, University of Vienna, Josef Holaubek Platz 2, A-1090 Vienna, Austria; 5 Department of Food Science, Faculty of Technical Sciences, Aarhus University, 8200 Aarhus, Denmark

**Keywords:** Mycotoxigenic fungi, biocontrol, *Artemisia* species, food safety, essential oils

## Abstract

Mycotoxigenic fungi, particularly species of *Aspergillus*, *Fusarium*, and *Penicillium*, contribute substantially to food contamination through the production of chemically diverse mycotoxins. Plant-derived essential oils (EOs) and extracts have therefore attracted increasing interest as potential alternatives or complements to conventional antifungal strategies. The genus *Artemisia*, which comprises several aromatic species with extensive biological properties, may constitute a promising source of such natural agents. This review critically examines the available data on the use of EOs and extracts from four *Artemisia* species (*A. absinthium*, *A. campestris*, *A. herba-alba*, and *A. judaica*) against mycotoxigenic fungi, with an emphasis on chemical composition, antifungal efficacy, mechanisms of action, and safety. Across the available studies, evidence suggests that *Artemisia* preparations frequently inhibit fungal growth, but their efficacy varies substantially according to species, geographical origin, plant part, harvesting conditions, extraction method, chemical composition, fungal species, and experimental assay. Importantly, evidence for direct inhibition of mycotoxin biosynthesis remains considerably more limited than evidence for fungal growth inhibition. Proposed antifungal mechanisms include disruption of the fungal cell wall and plasma membrane, altered mitochondrial function, and interference with cellular transport processes, although mechanistic evidence specifically for *Artemisia* preparations remains limited. Available toxicological data further indicate that efficacy and safety must be evaluated together, particularly for chemotypes containing potentially hazardous constituents such as thujone. Overall, *Artemisia* EOs and extracts may represent promising but not yet sufficiently validated candidates for food-associated mycotoxin control. Future studies could prioritize chemical standardization, mechanistic characterization, mycotoxin-specific endpoints, food-matrix validation, and integrated efficacy–safety assessment.

## 1. Introduction

Mycotoxigenic fungal species such as *Aspergillus*, *Fusarium*, and *Penicillium* been associated with adverse effects on agricultural productivity and the production of hazardous secondary metabolites, termed mycotoxins [1,2]. These highly toxic metabolites pose a significant threat to human and animal health because of their possible carcinogenicity, hepatotoxicity, mutagenicity, nephrotoxicity, and teratogenicity [2]. Major mycotoxins of human and animal health concern include aflatoxins (B1, B2, G1, G2), ochratoxin A (OTA), zearalenone, fumonisins (B1 and B2), trichothecenes such as T-2 toxin, HT-2, and deoxynivalenol [1]. Biological control approaches, such as the use of beneficial botanical extracts, are often preferred to inhibit the growth of mycotoxigenic fungi and detoxify mycotoxins, as they are considered environmentally friendly and safe [3]. Furthermore, the application of plant products, such as essential oils (EOs) and plant extracts, using an efficient delivery system, such as nano-delivery systems, provides an opportunity to avoid the risk associated with synthetic chemical preservatives and fungicides [4]. Recent investigations have examined several EOs, extracts, and their bioactive compounds for activity against mycotoxigenic fungal species and associated mycotoxins [4,5].

Within this context, the genus *Artemisia* belongs to the kingdom Plantae, class Magnoliopsida, order Asterales, and family Asteraceae and comprises over 500 species [6]. In general, *Artemisia* species are small shrubs or annual, perennial, and biennial herbs [6]. They have leaves of varying forms, silvery or grayish foliage, and inconspicuous white or yellow flowers with small black seeds. The species are generally recognized as aromatic plants capable of producing many EOs with diverse biological activities, including antioxidant, neuroprotective, hepatoprotective, anthelmintic, insecticidal, antiviral, and antifungal effects [7,8]. Several *Artemisia* species are predominantly distributed in temperate zones across Europe, Asia, North America, and Africa, and under favorable growth conditions, they may reach heights of 80–150 cm [9].

**Figure 1:**
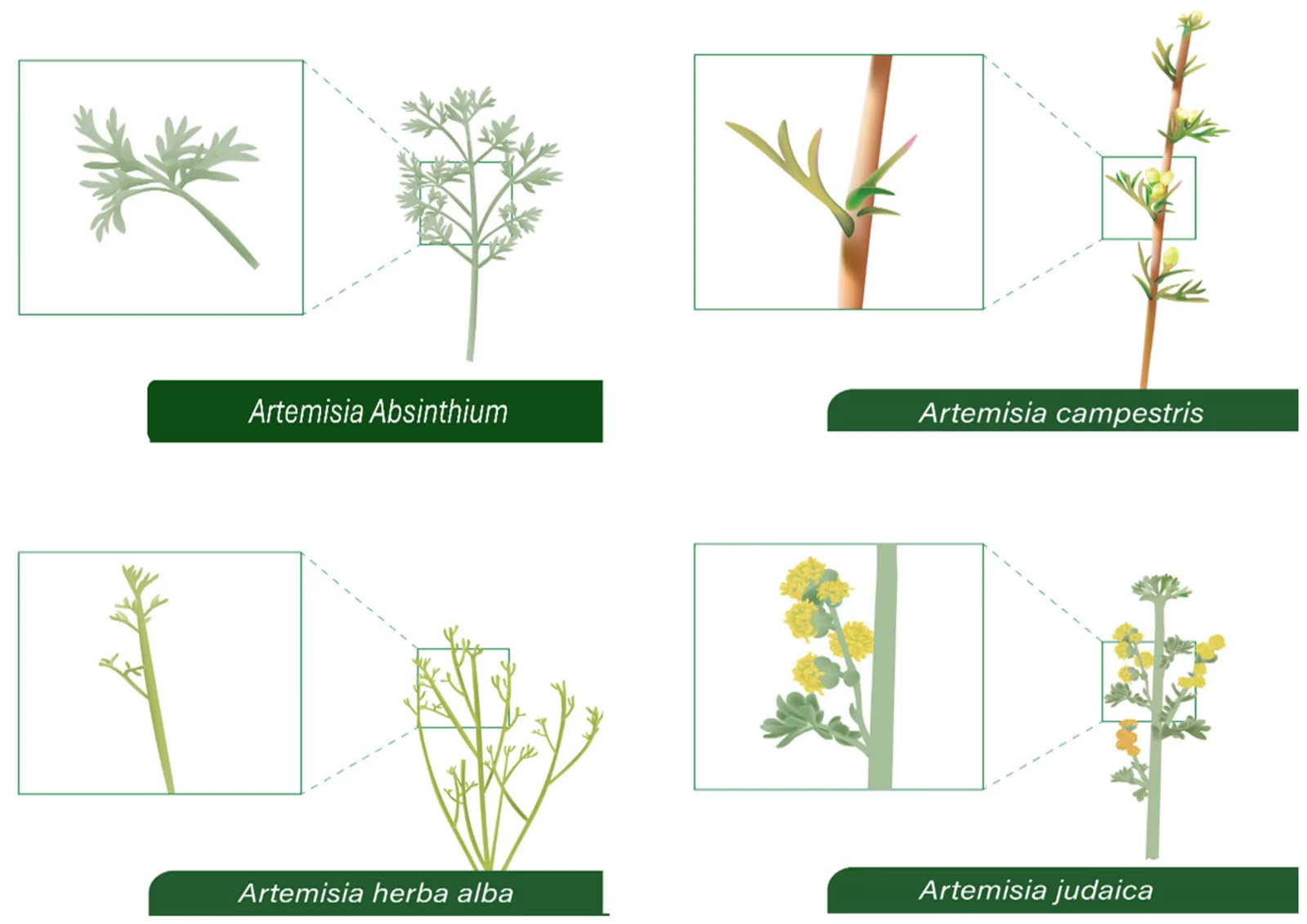
Morphological appearances of four different *Artemisia* species.

Among *Artemisia* species, four species appear to have been extensively studied for various potential applications, including limited activities relevant to toxigenic fungal control (Figure 1). *Artemisia absinthium*, commonly referred to as wormwood, is a yellow-flowering perennial plant distributed across Europe, the Middle East, North Africa, and Asia, and has been extensively studied for its diverse biological activities [10,11,12]. *Artemisia campestris*, commonly known as “Tgouft” or “armoise rouge”, is a perennial undershrub widely distributed across the Northern Hemisphere, particularly in arid regions of North Africa [13,14]. This species may exhibit various medicinal and antimicrobial activities [14,15]. *Artemisia herba-alba*, known as desert wormwood or white wormwood, is a medicinal and aromatic dwarf shrub that grows in arid and semi-arid regions of the Mediterranean, North Africa, and the Middle East [16]. As reported in the literature, its EOs and extracts have reportedly demonstrated various biological and pharmacological activities [13,17,18]. *Artemisia judaica*, a fragrant small shrub with pubescent leaves, is another noteworthy species with traditional use in treating various ailments [6,19]. This perennial plant is distributed in the southern deserts of Algeria, Egypt, and other Middle Eastern countries, such as Jordan and Saudi Arabia, and has been employed in traditional medicine [20].

The presence of mycotoxigenic fungi in food represents a significant concern, and the medicinal and antifungal potential of *Artemisia* species suggests that their EOs and extracts can be explored as natural alternatives to synthetic preservatives for food preservation and mycotoxin control. This review presents a summary of the potential of EOs and plant extracts from *A. absinthium*, *A. campestris*, *A. herba-alba*, and *A. judaica* to control mycotoxigenic fungi and their associated mycotoxins. In addition, subsequent sections describe the ecological and geographical conditions, harvesting stage, plant part, and extraction methodology that influence EO and extract composition and antifungal efficacy; outline proposed general mechanisms of action of plant extracts and EOs; and discuss the available data regarding the safety of *Artemisia* preparations. The four species covered in this review were selected based on predefined criteria rather than on a ranking of the genus as a whole. These criteria included their ethnopharmacological importance in the Mediterranean and Middle Eastern regions, where the available studies on *Artemisia* antifungal activity originate; the availability of published in vitro data regarding their antifungal activity; and their phytochemical characterization.

## 2. Literature Search Methodology and Overview of the Retrieved Studies

This review was conducted as a narrative review informed by a structured literature search. A total of 99 articles were identified across multiple databases (e.g., Web of Science, Scopus, PubMed, and Google Scholar) together with non-indexed papers from other scientific journals and publishers, for studies published between 1990 and 2025. The search combined the terms “*Artemisia*,” “essential oil,” “plant extract,” “antifungal,” “toxigenic fungi,” “mycotoxigenic fungi,” and “mycotoxin”. Only articles published in English were considered. Books, conference abstracts, and studies restricted to yeast species were excluded. Out of the 99 studies, 38 focused on elucidating the effects of EOs and extracts against several toxigenic fungi, including *Aspergillus (niger*, *fumigatus*, *ochraceus*, *versicolor*, *flavus*, *parasiticus*, and *oryzae)*, *Penicillium (expansum*, *citrinum*, *brevicompactum*, *viridicatum*, and *aurantiogriseum)*, *Fusarium (culmorum*, *graminearum*, *oxysporum*, *proliferatum*, *verticillioides*, *moniliforme*, *solani*, *acuminatum*, *equiseti*, and *sambucinum)*, and *Alternaria (alternata* and *solani).*

Among the 99 studies, 61 articles exclusively described the chemical composition of essential oils without investigating their antifungal activities. The studies were conducted across various countries worldwide, with the majority originating from Algeria (21), Tunisia (18), Egypt (12), and Morocco (9). Other countries contributing to the research include Saudi Arabia (4), Jordan (5), Bulgaria (2), China (2), Poland (2), Libya (2), Iran (2), Turkey (4), Brazil (2), Pakistan (2), Serbia (2), Canada (1), Ethiopia (1), India (1), Portugal (1), Tajikistan (1), Uruguay (1), Iraq (1), and Albania (1). Two studies did not mention the country of origin.

Of the 38 studies reviewed, various in vitro methods have been employed to investigate the antifungal properties of EOs and extracts from the *Artemisia* species. These methods include the paper disc diffusion method, agar well diffusion method, direct contact method, agar dilution method, cup plate agar diffusion, poisonous medium technique, mycelia growth inhibition assay, spore germination assay, tube dilution method, fumigation bioassay, and broth dilution assay. The findings were presented using various methods of expression, such as minimal inhibitory concentration (MIC), minimum fungicidal concentration (MFC), percentage inhibition of mycelia growth, effective and inhibitory concentration causing 50% mycelia growth inhibition (EC50), half maximal inhibitory concentration (IC50), medium inhibitory concentration (MIC50), and diameter inhibition zone.

## 3. Major EOs and Extracts from *Artemisia* Species Used Against Mycotoxigenic Fungi

### 3.1. EOs and Extracts from Artemisia absinthium

Within the genus *Artemisia*, *A. absinthium* appears to be the most studied species, with numerous EOs and extracts tested against mycotoxigenic fungi and fully characterized by analytical techniques such as gas chromatography with flame ionization detection (GC/FID) and gas chromatography with mass spectrometry (GC/MS) (Table 1). Major EO components include chamazulene, myrcene, trans-sabinyl acetate, cis-chrysanthenyl acetate, β-thujone, davanone, camphor, neryl isovalerate and geranyl isobutanoate, with marked variation in these profiles arising from differences in origin and plant part [21].

Most EOs and extracts have been evaluated against *Aspergillus*, *Alternaria*, *Fusarium*, and *Penicillium* species using disc diffusion, contact assays, and poisoned food techniques [22,23]. Tunisian EOs showed MICs of 12.5–25% (*v*/*v*) and MFCs of 25–50% (*v*/*v*) against *Aspergillus niger* and *Aspergillus flavus* [24], whereas Saudi EOs were active at 0.0025 and 0.1 mg/mL against *Aspergillus parasiticus*, *Aspergillus niger*, and *Aspergillus flavus* [25,26] and Indian EOs at 0.183 and 0.091 mg/mL against *Aspergillus niger* and *Aspergillus fumigatus*, respectively [27]. Turkish EOs inhibited numerous *Aspergillus* and *Fusarium* strains with 8–85% inhibition depending on dose, though *Alternaria alternata* and *F. solani* were less sensitive at lower doses [22,23]. Canadian EOs from aerial parts produced inhibition zones of 18 mm against *Aspergillus niger* at 10 µL [28]. Collectively, these findings suggest both the broad antifungal potential of *A. absinthium* EOs and the strong influence of origin and chemotype on potency.

Extracts appeared to exhibit greater variability than EOs. Some methanolic and aqueous preparations from Turkish *A. absinthium* leaves, stems, and flowers, using Soxhlet extraction and maceration, do not significantly affect *Aspergillus niger* [29], whereas methanolic extracts rich in caffeoylquinic acids and luteolin derivatives inhibit *F. culmorum* (35.2%) and *F. graminearum* (10%) at 60 mg/mL [30]. Aqueous leaf extracts appeared to exert substantial inhibition of *Alternaria alternata* (79.7%) and *P. expansum* (75.4%) at 1000 mg/mL after seven days of incubation at 24 °C. Later work demonstrated inhibition of *Aspergillus niger*, *Aspergillus flavus*, and *P. expansum*, with the highest activity against *P. expansum* (75%), followed by *Aspergillus flavus* (74.7%) and *Aspergillus niger* (61.9%) at the same concentration and incubation time. The corresponding EOs reduced spore germination of all three fungi, with stronger effects on *Aspergillus niger* (70.7%) than on *Aspergillus flavus* (40.7%) and *P. expansum* (43.3%) [31,32]. Hydroethanolic leaf extracts from Pakistan produced 75.4% and 77.5% growth inhibition at 2 mg/mL for *Aspergillus flavus* and *F. oxysporum*, respectively [33], and different plant parts and solvents yielded varying sensitivity profiles, with *F. oxysporum* particularly responsive to ethanol extracts from leaves, flowers, stems, and roots providing more active ethyl acetate extracts [34].

These results suggest that *A. absinthium* EOs may provide broad-spectrum inhibition of mycotoxigenic *Aspergillus*, *Fusarium*, *Alternaria*, and *Penicillium* spp., while extracts offer additional but more variable activity at the in vitro scale. However, most studies emphasize growth inhibition rather than mycotoxin production, and substantial chemotypic diversity makes it challenging to define universally effective preparations for mycotoxin control, highlighting the need for integrated studies that link EO composition, fungal inhibition and mycotoxin outcomes.

**Table 1:** Antifungal activity of *Artemisia absinthium* against different fungal species.

Plant Part	EOs/Extract	Extraction Methods	Country	Major Components (%)	Fungal Species	Testing Conditions	Observed Effect	References
Aerial parts	EOs	Hydro distillation	Turkey	N.A.	*A. niger*	Contact assay (20 µL)	Growth inhibition (76.5%)	[23]
					*A. alternata*		Growth inhibition (20%)	
					*A. solani*		Growth inhibition (61%)	
					*F. graminearum*		Growth inhibition (85.3%)	
					*F. oxysporum*		Growth inhibition (35%)	
					*F. proliferatum*		Growth inhibition (77.9%)	
					*F. solani*		Growth inhibition (13.7%)	
					*F. verticillioides*		Growth inhibition (78.3%)	
					*F. culmorum*		Growth inhibition (82.2%)	
Aerial parts	EOs	Hydro distillation	Turkey	Chamazulene (17.8) Nuciferol butanoate (8.2) Nuciferol propionate (5.1)	*F. oxysporum*	Contact assay (10, 20, and 40 µL)	Growth inhibition (41.9%) Growth inhibition (47.7%) Growth inhibition (51.5%)	[22]
					*F. solani*		No effect Growth inhibition (18.6%) Growth inhibition (14.2%)	
					*F. sambucinum*		Growth inhibition (32.5%) Growth inhibition (38.5%) Growth inhibition (52.4%)	
					*Penicillium* sp.		Growth inhibition (8.1%) Growth inhibition (70.5%) Growth inhibition (26.6%)	
					*A. alternata*		No effect Growth inhibition (13.3%) Growth inhibition (50.7%)	
Aerial parts	EOs	Hydro distillation	Serbia (Mokra)	*β*-thujone (19.8) cis-*β*-epoxyocimene (10.7) Trans-sabinyl acetate (8.8)	*A. niger*	Paper disc diffusion (1:10 and 1:30) (50 µL)	IZ diameter = 25 mm IZ diameter = 16 mm	[35]
				*β*-thujone (20.2) 1,8-cineole (16.3) Trans-sabinyl acetate (15.5)			IZ diameter = 29 mm IZ diameter = 18 mm	
			Serbia (Nis)	β-thujone (63.4) sabinene (10.8) *p*-cymene (6.7)			IZ diameter = 31 mm IZ diameter = 17 mm	
Aerial parts	EOs	Hydro distillation	Canada	Trans-sabinyl acetate (26.4) Myrcene (10.8) Trans-thujone (10.1)	*A. niger*	Drop agar diffusion (10 µL)	IZ diameter 18 mm	[28]
Aerial parts	EOs	Hydro distillation	Saudi Arabia	Cis-davanone (34.7) Camphor (16.2) Chamazulene (8.2)	*A. parasiticus*	Broth microdilution	MIC = 0.0025 mg/mL	[26]
Aerial parts	EOs	Soxhlet Extraction	Saudi Arabia	N.A.	*A. niger*	Broth microdilution	MIC = 0.1 mg/mL	[25]
					*A. flavus*		MIC = 0.1 mg/mL	
					*A. niger*	Agar well diffusion	IZ diameter = 21 mm	
					*A. flavus*		IZ diameter = 16 mm	
Leaves, stems, and flowers	Methanolic extract	Soxhlet Extraction	Turkey	N.A.	*A. niger*	Paper disc diffusion (0.3 mg/disc)	No effect	[29]
	Aqueous extract	Maceration					No effect	
Leaves, stems, and flowers	EOs	Hydro distillation	Tunisia	Chamazulene (34.8) Trans-Sabinene hydrate (21.7) β-thujone (6.7)	*F. culmorum*	Paper disc diffusion (20 µL/disc)	Growth inhibition (46.8%)	[36]
					*F. graminearum*		Growth inhibition (16.7%)	
					*F. oxysporum*		Growth inhibition (25.3%)	
				Chamazulene (29.5) β-thujone (22.0) trans-Sabinene hydrate (12.0)	*F. culmorum*		Growth inhibition (42%)	
					*F. graminearum*		Growth inhibition (11%)	
					*F. oxysporum*		Growth inhibition (17.9%)	
				Chamazulene (25.3) β-thujone (21.0) trans-Sabinene hydrate (12.5)	*F. culmorum*		Growth inhibition (45.2%)	
					*F. graminearum*		Growth inhibition (14.7%)	
					*F. oxysporum*		Growth inhibition (19%)	
				Chamazulene (39.9) β-thujone (17.1) Trans-Sabinene hydrate (12.0)	*F. culmorum*		Growth inhibition (23.7%)	
					*F. graminearum*		Growth inhibition (23.7%)	
					*F. oxysporum*		Growth inhibition (27.6%)	
Leaves and stems	Methanolic extract	Maceration	Algeria	5-caffeoylquinic acid (23.0) Glucosyl–arabinosyl–luteolin (20.5) Dicaffeoylquinic acid (21.1) 3-caffeoylquinic acid (6.4) 4-caffeoylquinic acid (4.4) Luteolin rutinoside (3.8)	*F. culmorum*	Poisonous medium technique (60 mg/mL)	Growth inhibition (35.2%)	[30]
					*F. graminearum*		Growth inhibition (10%)	
Leaves	EOs	Hydro distillation	India	Borneol (18.7) methyl hinokiate (12.9)	*A. niger*	Tube dilution (0.009 to 5 mg/mL)	MIC = 0.183 mg/mL	[27]
					*A. fumigatus*		MIC = 0.091 mg/mL	
Leaves	Aqueous extract	N.A.	N.A.	N.A.	*A. alternata*	Contact assay (1000 mg/mL)	Growth inhibition (79.7%)	[31]
					*P. expansum*		Growth inhibition (75.4%)	
Leaves	EOs	Hydro distillation	Tunisia (Ghar Dimaou, El Kef, Kasserine, Gafsa)	Camphor (50.3) (Z)-sabinene hydrate (11.8) 1-terpinen-4-ol (6.8)	*A. niger*	Broth dilution	MIC = 12.5% (*v*/*v*) MFC = 25% (*v*/*v*)	[24]
				Camphor (28.9) Bornyl acetate (15.5) Chamazulene (18.9)	*A. flavus*		MIC = 25% (*v*/*v*) MFC = 50% (*v*/*v*)	
				Camphor (33.2) Chamazulene (24.6) Linalool (6.4)	*A. niger*	Paper disc diffusion	IZ diameter = 15 mm	
							IZ diameter = 15 mm	
							IZ diameter = 16 mm	
							IZ diameter = 14 mm	
				Chamazulene (42.9) Camphor (12.9)	*A. flavus*		IZ diameter = 12 mm	
							IZ diameter = 12 mm	
							IZ diameter = 13 mm	
							IZ diameter = 13 mm	
Leaves	Aqueous extract	N.A.	N.A.	N.A.	*A. niger*	Contact assay (1000 mg/mL)	Growth inhibition (61.9%)	[32]
					*A. flavus*		Growth inhibition (74.7%)	
					*P. expansum*		Growth inhibition (75%)	
					*A. niger*	Spore germination assay (1000 mg/mL)	Growth inhibition (70.7%)	
					*A. flavus*		Growth inhibition (40.7%)	
					*P. expansum*		Growth inhibition (43.3%)	
Leaves	Ethyl acetate extract	Maceration	China	N.A.	*F. oxysporum*	Contact assay (0.5 mg/mL)	Growth inhibition (42.7%)	[34]
	Ethanolic extract						Growth inhibition (62.7%)	
	Aqueous extract						Growth inhibition (51.3%)	
Leaves	EOs	Hydro distillation	Pakistan	Oleic acid (41.4) 5-(hydroxymethyl)-2-Furancarboxaldehyde (14.2) 9,12-Octadecadienoic acid (Z, Z)-, Methyl ester (9.0) Estra-1,3,5(10) -trien-17β-ol (8.7) 9,9-Dimethoxybicyclo [3.3.1] nona-2,4-dione (7.7)	*A. niger*	Agar dilution (0.5 mg/mL)	Growth inhibition (70%)	[37]
					*A. flavus*		Growth inhibition (67%)	
Leaves	Hydro ethanolic extract	N.A.	Pakistan	N.A.	*A. flavus*	Poisoned food technique (2 mg/mL)	Growth inhibition (75.4%)	[33]
					*F. oxysporum*		Growth inhibition (77.5%)	
Flowers	Ethyl acetate extract	Maceration	China	N.A.	*F. oxysporum*	Contact assay (0.5 mg/mL)	Growth inhibition (45.8%)	[34]
	Ethanolic extract						Growth inhibition (65.7%)	
	Aqueous extract						Growth inhibition (53.4%)	
Stems	Ethyl acetate extract	Maceration	China	N.A.	*F. oxysporum*	Contact assay (0.5 mg/mL)	Growth inhibition (38.4%)	
	Ethanolic extract						Growth inhibition (42.7%)	
	Aqueous extract						Growth inhibition (38.9%)	
Roots	Ethyl acetate extract	Maceration	China	N.A.	*F. oxysporum*	Contact assay (0.5 mg/mL)	Growth inhibition (72.5%)	
	Ethanolic extract						Growth inhibition (64.6%)	
	Aqueous extract						Growth inhibition (47.9%)	
Roots	EOs	Hydro distillation	Serbia (Nis)	α-fenchene (23.3) Linalyl 3-methylbutanoate (21.1) Linalyl butanoate (14.4)	*A. niger*	Paper disc diffusion (1:10 and 1:30 dilution) (50 µL)	IZ diameter = 24 mm IZ diameter = 15 mm	[35]

EOs: Essential Oils, MIC: Minimum Inhibitory Concentration, MFC: Minimum Fungicidal Concentration, IZ: Inhibition Zone, N.A: Not Available. Values are reported as in the original studies, with the original units and endpoints. Because the studies used different experimental methods, numerical values obtained with different endpoints or units are not directly comparable.

### 3.2. EOs and Extracts from Artemisia campestris

Compared with the other *Artemisia* species considered in this review, *A. campestris* has been investigated less frequently against mycotoxigenic fungi, and available data are mainly limited to methanolic extracts and hydrodistilled EOs from aerial and stem parts (Table 2). Methanolic stem extracts have been tested against six pathogenic and toxicogenic fungal species (*P. expansum*, *P. brevicompactum*, *F. oxysporum*, *F. verticillioides*, *Aspergillus fumigatus*, and *Aspergillus flavus*), with the highest inhibition observed for *F. oxysporum* (54.4%), followed by *Aspergillus fumigatus* (47.4%), *F. verticillioides* (39.5%), *P. brevicompactum* (31.2%), and *Aspergillus flavus* (30.5%), while *P. expansum* showed only modest inhibition (6.7%) all at a concentration of 2 mg/mL [38]. These results suggest that *Fusarium* species, particularly *F. oxysporum*, are more sensitive to *A. campestris* stem extracts than some *Penicillium* and *Aspergillus* isolates.

Extraction methodology clearly influences antifungal performance. Methanol extracts obtained through classical extraction yield marginally larger inhibition zones against *Aspergillus niger* than those obtained by ultrasonic extraction (33.1 vs. 32.5 mm at 20 mg/mL), indicating that sonication may slightly reduce extract efficacy [15]. This reduction has been attributed to a possible degradation or oxidation of bioactive compounds through interactions with hydroxyl radicals generated during ultrasound treatment, especially under prolonged exposure [39]. Furthermore, Soxhlet-derived methanolic extracts from aerial parts inhibited *P. expansum* in several assay formats: 52% inhibition of mycelial growth at 400 µg/mL in contact assays, 16.1% inhibition at 200 mg/mL in agar well diffusion, and an inhibition-zone diameter of 16.42 mm at 800 mg/mL in disc diffusion assays. Although each format uses a different endpoint and concentration scale, the results suggest, in aggregate, that the extract exhibits activity against *P. expansum* and that, within each format, the observed effect increased with concentration. Hydrodistilled EOs from aerial parts of *A. campestris* appear to exhibit substantial antifungal activity, particularly against *P. expansum*. Depending on the assay, the applied EOs inhibited *P. expansum* with an inhibition-zone diameter of 59 mm in disc diffusion assays (15 µL/disc), 49.7% inhibition of mycelial growth in fumigation assays (20 µL), and 81.4% inhibition in contact assays (0.8 µL/mL) [40]. Additional studies suggest that *A. campestris* EOs can inhibit other mycotoxigenic fungi, a finding that may support their classification as effective antifungal agents [41,42]. The chemical composition of hydrodistilled EOs from *A. campestris* has been characterized by GC and GC–MS, revealing terpenoid-rich profiles dominated by monoterpenes and sesquiterpenes. Major constituents commonly include α-pinene, β-pinene, p-cymene, camphor, germacrene D, and spathulenol, although the relative abundance of these compounds varies among countries and plant parts [43,44].

**Table 2:** Antifungal activity of *Artemisia campestris* against different fungal species.

Plant Part	EOs/Extract	Extraction Methods	Country	Major Components (%)	Tested Fungal Species	Testing Conditions	Observed Effect	References
Aerial parts	Methanolic extract	Conventional extraction	Bulgaria	N.A.	*A. niger*	Agar well diffusion (20 mg/mL)	IZ diameter = 33.1 mm	[15]
		Ultrasound extraction					IZ diameter = 32.5 mm	
Aerial parts	EOs	Hydro distillation	Algeria	N.A.	*P. expansum*	Micro-well dilution	MIC = 2.5 mg/mL MFC = 2.5 mg/mL	[40]
						Fumigation bioassay (20 µL)	Growth inhibition (49.7%)	
						Contact assay (0.8 µL/mL)	Growth inhibition (81.4%)	
						Paper disc diffusion (15 μL/disc)	IZ diameter = 59 mm	
	Methanolic extract	Soxhlet extraction				Contact assay (0.4 mg/mL)	Growth inhibition (52%)	
						Agar well diffusion (200 mg/mL)	Growth inhibition (16.1%)	
						Paper disc diffusion (800 mg/mL)	IZ diameter = 16.42 mm	
Aerial parts	EOs	Hydro distillation	Portugal	β-pinene (54.5) Cadin-4-en-7-ol (9.5) (Z)-β-ocimene (6.0) γ-terpinene (4.6)	*A. niger*	Broth macrodilution	MIC = 2.5 mg/mL MFC = 10 mg/mL	[42]
					*A. fumigatus*		MIC = 10 mg/mL MFC = 10 mg/mL	
					*A. flavus*		MIC = 10 mg/mL MFC = 10 mg/mL	
Aerial parts	EOs	Hydro distillation	Algeria	N.A.	*A. niger* *P. viridicatum*	Broth macrodilution	MIC = 10 mg/mL MFC = 20 mg/mL	[41]
					*A. ochraceus* *A. parasiticus*		MIC = 2.5 mg/mL MFC = 5 mg/mL	
					*A. flavus* *P. expansum* *F. culmorum* *F. moniliforme*		MIC = 2.5 mg/mL MFC = 2.5 mg/mL	
					*P. citrinum*		MIC = 5 mg/mL MFC = 20 mg/mL	
					*F. graminearum*		MIC = 1.25 mg/mL MFC = 1.25 mg/mL	
Stems	Methanolic extract	Maceration	Bulgaria	N.A.	*A. fumigatus*	Agar dilution (2 mg/mL)	Growth inhibition (47.4%)	[38]
					*A. flavus*		Growth inhibition (30.5%)	
					*P. expansum*		Growth inhibition (6.7%)	
					*F. verticillioides*		Growth inhibition (39.5%)	
					*F. oxysporum*		Growth inhibition (54.4%)	
					*P. brevicompactum*		Growth inhibition (31.2%)	

EOs: Essential Oils, MIC: Minimum Inhibitory Concentration, MFC: Minimum Fungicidal Concentration, IZ: Inhibition Zone, N.A: Not Available. Values are reported as in the original studies, with the original units and endpoints. Because the studies used different experimental methods, numerical values obtained with different endpoints or units are not directly comparable.

### 3.3. EOs and Extracts from Artemisia herba-alba

Multiple in vitro studies have investigated the efficacy of *A. herba-alba* against mycotoxigenic fungi, particularly *Aspergillus* and *Penicillium* species, which are common contaminants associated with food spoilage (Table 3). Most work has examined EOs and crude extracts from aerial parts using agar well diffusion and related assays, and only one study has directly assessed antimycotoxigenic activity [45]. Leaf EOs from *A. herba-alba* exhibited complete inhibition of mycelial growth of *P. aurantiogriseum* and *P. viridicatum* at concentrations of 0.89% (*v*/*v*) and 1.33% (*v*/*v*), respectively. At a concentration of 0.44% (*v*/*v*), the levels of penicillic acid, terrestric acid, aurantiamine, brevianamide A, and xanthomegnin were below the limit of detection of the qualitative thin-layer chromatography analysis performed in the assay [45]. These findings suggest that mycotoxin production may have been inhibited. Steam-distilled EOs from *A. herba-alba* appeared to achieve complete inhibition of *P. citrinum* growth at 1000 μg/mL [46], demonstrating that *A. herba-alba* EOs can affect both fungal growth and mycotoxin output in *Penicillium* spp.

EO composition and antifungal activity are substantially shaped by harvest season and geographic origin, which can induce variations in their chemical composition and biological properties. Moroccan EOs collected at different periods (March, June, and September) have been reported to exhibit MIC values as low as 0.03% (*v*/*v*) against *Aspergillus niger*, *P. expansum* and *P. digitatum*, consistent with high contents of oxygenated monoterpenes [47]. Libyan, Jordanian, Saudi, Algerian, Tunisian, Moroccan and Iraqi populations present distinct chemotypes (such as chrysanthenone/cis chrysanthenyl acetate/β-thujone; α-thujone/1,8-cineole/camphor; piperitone-rich profiles; capillene/2,4-pentadiynylbenzene) which may produce clear inhibition of *Aspergillus*, *Fusarium* and *Penicillium* spp. in diffusion assays [17,48,49,50,51,52,53]. Isolated piperitone and carvone also show potent activity against *P. citrinum* [46]. Extracts provide a heterogeneous pattern as methanolic and aqueous aerial-part extracts may inhibit *Aspergillus ochraceus*, *F. graminearum* and *P. expansum* (>13% at 100 mg/mL), yet some root and leaf extracts in methanol or water show no activity against *Aspergillus niger* [53,54]. Ethanolic leaf and stem extracts moderately inhibit *Aspergillus niger*, *Aspergillus flavus*, *Aspergillus oryzae*, *P. citrinum*, and *Alternaria alternata* (MIC 3–6%; MFC 6–10%), with *Aspergillus niger* and *P. citrinum* requiring the highest fungicidal concentration (MFC 10%) [55].

Overall, *A. herba-alba* EOs, especially oxygenated monoterpene-rich chemotypes from specific regions and seasons, show clear activity against key mycotoxigenic *Aspergillus*, *Penicillium*, and *Fusarium* species and may effectively suppress mycotoxin production in some *Penicillium* strains, but antimycotoxigenic evidence is still limited and highly dependent on chemotype and extraction conditions.

**Table 3:** Antifungal activity of *Artemisia herba-alba* against different fungal species.

Plant Part	EOs/Extract	Extraction Methods	Country	Major Components (%)	Tested Fungal Species	Testing Conditions	Observed Effect	References
Aerial parts	EOs	Hydro distillation	Tunisia	Cis-chrysanthenyl acetate (10.6) Sabinyl acetate (9.1) α-thujone (8.7)	*Fusarium* sp.	Paper disc diffusion (10 µL)	IZ diameter = 27 mm	[52]
					*F. solani*		IZ diameter = 27.7 mm	
Aerial parts	EOs	Hydro distillation	Jordan	β-thujone (25.1) α-thujone (22.9) 1,8-Cineole (20.1) Camphor (10.5)	*A. niger*	Broth macrodilution	MIC = 1.25 mg/mL MFC = 5 mg/mL	[49]
					*A. fumigatus*		MIC = 2.5 mg/mL MFC = 5 mg/mL	
Aerial parts	EOs	Hydro distillation	Libya	Chrysanthenone (20.8) Cis-chrysanthenyl acetate (17.6) Cis-thujone (13.6)	*A. niger*	Broth microdilution	MIC = 0.250 mg/mL MFC = 0.250 mg/mL	[48]
					*A. fumigatus*		MIC = 0.125 mg/mL MFC = 0.250 mg/mL	
					*A. ochraceus*		MIC = 0.060 mg/mL MFC = 0.125 mg/mL	
					*A. versicolor*		MIC = 0.060 mg/mL MFC = 0.125 mg/mL	
					*F. solani*		MIC = 0.75% (*v*/*v*)	
					*F. oxysporum*		MIC = 0.5% (*v*/*v*)	
Aerial parts	EOs	Hydro distillation	Morocco	Cis-thujone (25.5) Trans-thujone (17.7) Vanillyl alcohol (11.5)	*A. niger*	Agar well diffusion (20 µL)	IZ diameter = 23.6 mm	[17]
Aerial parts	EOs	Hydro distillation	Algeria	Chrysanthenone (24.1) Camphor (16.2) α-thujone (12.8) 1,8-Cineole (9.3)	*A. ochraceus*	Agar dilution	MIC = 0.005 mg/mL	[51]
					*P. expansum*		MIC = 0.0005 mg/mL	
					*F. oxysporum*		MIC = 0.0005 mg/mL	
Aerial parts	Aqueous extract	Maceration	Algeria	N.A.	*A. flavus*	Agar well diffusion (100 mg/mL)	No effect	[54]
					*A. parasiticus*		No effect	
					*A. ochraceus*		Growth inhibition (22.5%)	
					*F. graminearum*		Growth inhibition (13.2%)	
					*P. expansum*		Growth inhibition (26.9%)	
	Methanolic extract				*A. flavus*		No effect	
					*A. parasiticus*		No effect	
					*A. ochraceus*		Growth inhibition (29.3%)	
					*F. graminearum*		Growth inhibition (21.1%)	
					*P. expansum*		Growth inhibition (20.8%)	
Leaves, stems, and flowers	EOs	Hydro distillation	Saudi Arabia	Piperitone (44.6) (E)-Ethyl Cinnamate (14.7)	*A. fumigatus*	Cup-plate agar diffusion 20 mg/mL (50 µL)	IZ diameter = 20.6 mm	[50]
						Broth microdilution	MIC = 0.0039 mg/mL	
Leaves and stems	EOs	Hydro distillation	Morocco	Cis-chrysanthenyl acetate (27.6–30) β-thujone (15.4–23.2) α-thujone (3–5.4) Camphor (9.76–15.8) Sesquiterpenic davanone (1.4–3.9)	*A. niger*	Agar dilution (0.02 to 1% (*v*/*v*))	MIC = 0.03–0.4%	[47]
					*P. expansum*			
Leaves and stems	Ethanolic extract	N.A.	Egypt	N.A.	*A. niger*	Serial dilution (1.5 to 10% (*w*/*v*))	MIC = 6.0% MFC = 10.0%	[55]
					*A. flavus*		MIC = 3.0% MFC = 6.0%	
					*A. oryzae*		MIC = 3.0% MFC = 6.0%	
					*A. alternata*		MIC = 6.0% MFC = 6.0%	
					*P. citrinum*		MIC = 3.0% MFC = 10.0%	
Leaves	EOs	Hydro distillation	Morocco	β-thujone (35.9) α-thujone (22.4) Camphor (13.9)	*P. aurantiogriseum*	Mycelia dry weight assay (0.89% (*v*/*v*))	Growth inhibition (100%)	[45]
					*P. viridicatum*	Mycelia dry weight assay (1.33% (*v*/*v*))	Growth inhibition (100%)	
Leaves	EOs	Steam distillation	Egypt	Carvone piperitone	*P. citrinum*	Mycelia dry weight assay (1 mg/mL)	Growth inhibition (100%)	[46]
	Piperitone					Mycelia dry weight assay (0.001 to 1 mg/mL)	IC50 = 0.002 mg/mL	
	Carvone						IC50 = 0.005 mg/mL	
Leaves	EOs	Hydro distillation	Iraq	2,4-pentadiynylbenzene (27.3) Capillene (24.3) 2-Methylnaphthalene (8.5)	*A. niger*	Agar well diffusion (50 µL)	IZ diameter = 53 mm	[53]
Leaves	Methanolic extract	Maceration		N.A.			No effect	
Leaves	Aqueous extract						No effect	
Roots	Methanolic extract						No effect	

EOs: Essential Oils, IC50: Concentration causing 50% inhibition, MIC: Minimum Inhibitory Concentration, MFC: Minimum Fungicidal Concentration, IZ: Inhibition Zone, N.A: Not Available. Values are reported as in the original studies, with the original units and endpoints. Because the studies used different experimental methods, numerical values obtained with different endpoints or units are not directly comparable.

### 3.4. EOs and Extracts from Artemisia judaica

*Artemisia judaica* has attracted moderate attention for antifungal properties, but studies specifically targeting mycotoxigenic fungi remain limited and focus mainly on EOs. Tested species include *Aspergillus* (*Aspergillus niger*, *Aspergillus versicolor*, *Aspergillus ochraceus*, *Aspergillus fumigatus*), *Fusarium* (*F. solani*, *F. oxysporum*), and *Alternaria alternata*, while aflatoxigenic *Aspergillus flavus* and *Aspergillus parasiticus* have not yet been evaluated. EOs from parts of *A. judaica*, such as aerial parts, leaves, stems and flowers, show their most consistent activity against *Aspergillus niger*, *Aspergillus fumigatus*, *Aspergillus ochraceus*, and *Aspergillus versicolor* with moderate to strong inhibitory activity and low MIC values in some chemotypes and measurable fungicidal effects, supported by clear inhibition zones in disc diffusion assays (Table 4). This activity is closely linked to the predominance of oxygenated monoterpenes. In particular, piperitone-rich chemotypes have been repeatedly associated with enhanced activity [48]. Mechanistic findings from related systems indicate that such oxygenated monoterpenes disrupt fungal membrane integrity, increase membrane permeability, and induce oxidative stress through reactive oxygen species (ROS) accumulation, and piperitone can suppress aflatoxin biosynthetic gene expression in *Aspergillus flavus* [56], although this effect has not yet been validated specifically using *A. judaica* EO. Other constituents such as β-thujone, terpinene-4-ol, thymol, and carvacrol likely contribute synergistically, reflecting a multi-target mode of action typical of complex EO matrices. Substantial compositional variability has been reported across geographic origins (North Africa and the Arabian Peninsula). Differences in dominant constituents, such as piperitone-, β-thujone-, or sesquiterpene-rich chemotypes, correlate with differences in antifungal potency, which may underscore the influence of ecological and environmental factors on bioactivity. Against *Fusarium* spp., *A. judaica* EOs exhibit more variable activity. While some chemotypes demonstrate strong inhibition of spore germination, suppression of mycelial growth is generally moderate and dose-dependent. The activity may be related to the ability of monoterpenes to induce lipid peroxidation and oxidative cellular damage, ultimately leading to structural membrane disruption. However, compared with *Aspergillus* spp., *Fusarium* isolates often require higher concentrations to achieve comparable inhibitory effects, suggesting genus-specific differences in susceptibility. Similarly, *Alternaria alternata* is even less sensitive, requiring higher EO concentrations for only moderate inhibition. Given the agricultural relevance of *Alternaria*-associated mycotoxins, this area remains insufficiently explored.

Across studies, extraction techniques and chemotypes strongly influenced antifungal performance [57]. Hydro-distilled oils usually exhibited stronger inhibition than those from microwave-assisted or alternative extraction methods, likely due to differences in thermal stability and recovery of oxygenated monoterpenes [58]. Chemotypic variation is affected by geographic location, harvesting stage, environmental conditions, and post-harvest processing, which collectively modulate secondary metabolite profiles, ultimately shaping biological efficacy [59,60]. Such variability complicates cross-study comparison and highlights the need for standardized extraction protocols and comprehensive chemical profiling when evaluating antifungal activity [61]. In contrast, solvent extracts (organic-based or aqueous) of *A. judaica* show inconsistent and generally weaker antifungal effects. Non-polar and mid-polar extracts often lacked measurable activity against *Aspergillus niger*, whereas methanol and ethyl acetate extracts occasionally produced modest inhibition zones [62]. Some extracts showed higher activity against various *Fusarium* species than against *Aspergillus* species (Table 4). A major limitation of extract-based studies is the frequent absence of chemical characterization, preventing identification of the active metabolites responsible for observed activity [63]. Consequently, in the absence of phytochemical profiling, antifungal effects cannot be unambiguously attributed to sesquiterpene lactones, flavonoids, phenolic acids, or alternative secondary metabolites.

**Table 4:** Antifungal activity of *Artemisia judaica* against different fungal species.

Plant Part	EOs/Extract	Extraction Methods	Country	Major Components (%)	Fungal Species	Testing Conditions	Observed Effect	References
Aerial parts	EOs	Hydro distillation	Egypt	β-thujone (49.8) Chrysanthenone (10.8) α-thujone (8.2)	*A. alternata*	Mycelial growth inhibition	EC50 = 0.54 mg/mL	[64]
						Spore germination assay	EC50 = 0.20 mg/mL	
					*F. oxysporum*	Mycelial growth inhibition	EC50 = 0.73 mg/mL	
						Spore germination assay	EC50 = 0.06 mg/mL	
					*F. solani*	Mycelial growth inhibition	EC50 = 0.86 mg/mL	
						Spore germination assay	EC50 = 0.45 mg/mL	
Aerial parts	EOs	Hydro distillation	Libya	Piperitone (30.2) Cis-Chrysanthenol (9.1)	*A. niger*	Broth microdilution	MIC = 0.06 mg/mL MFC = 0.12 mg/mL	[48]
					*A. fumigatus*		MIC = 0.12 mg/mL MFC = 0.20 mg/mL	
					*A. ochraceus*		MIC = 0.06 mg/mL MFC = 0.12 mg/mL	
					*A. versicolor*		MIC = 0.03 mg/mL MFC = 0.06 mg/mL	
Aerial parts	EOs	Hydro distillation	Jordan	Piperitone (30.4) Camphor (16.1) (E)-Ethyl Cinnamate (11.0)	*A. niger*	Broth macrodilution	MIC = 0.6 mg/mL MFC = 5 mg/mL	[65]
					*A. fumigatus*		MIC = 0.6 mg/mL MFC = 5 mg/mL	
Aerial parts	Petroleum ether extract	Maceration	Egypt	N.A.	*A. niger*	Paper disc diffusion	No effect	[62]
					*A. fumigatus*		No effect	
	Methylene chloride extract				*A. niger*		No effect	
					*A. fumigatus*		IZ diameter = 7 mm	
	Ethyl acetate extract				*A. niger*		IZ diameter = 8 mm	
					*A. fumigatus*		No effect	
	Acetone extract				*A. niger*		No effect	
					*A. fumigatus*		No effect	
	Methyl alcohol extract				*A. niger*		IZ diameter = 8 mm	
					*A. fumigatus*		No effect	
Aerial parts	EOs	Hydro distillation	Saudi Arabia	Piperitone (31.9) Trans-Ethyl Cinnamate (13.6) Terpinene-4-ol (6.4) α-thujone (5.9)	*A. niger*	Paper disc diffusion (20 µL)	IZ diameter = 15 mm	[20]
						Broth microdilution	MIC = 6.25 mg/mL MFC = 12.5 mg/mL	
Aerial parts	Methanolic extract	Soxhlet extraction	N.A.	Kaempferol (12.2) Apigenin (30.5) Chlorogenic acid (3.6)	*A. niger*	Agar well diffusion assay (100 µL)	No effect	[66]
						Broth microdilution		
Leaves, stems, and flowers	EOs	Hydro distillation	Saudi Arabia	*β*-eudesmol (13.1) Hexadecanoic acid (5.7) Spathulenol (3.7) Eudesma-4 (15),7-dien-1-ol (3.5) Carvacrol (3.5) Thymol (3.5)	*A. fumigatus*	Cup-plate agar diffusion (20 mg/mL in 50 µL)	IZ diameter = 16.8 mm	[50]
						Broth microdilution	MIC = 0.03 mg/mL	
Leaves, stems, and flowers	EOs	Hydro distillation	Egypt	Piperitone (33) 3-Bornanone (28.5) p-Cymen-8-ol (5.5) 1-Borneol (4.8) Isophorone (3.8) Camphene (2.2)	*A. niger*	Paper disc diffusion (10 μL/disc)	IZ diameter = 15 mm	[58]
						Paper disc diffusion (0.15 to 5.0 μL/disc)	MIC = 1.25 μL/disc	
					*F. solani*	Paper disc diffusion (10 μL/disc)	IZ diameter = 12 mm	
						Paper disc diffusion (0.15 to 5.0 μL/disc)	MIC = 2.5 μL/disc	
		Microwave distillation		Piperitone (34) 3-Bornanone (32.8) p-Cymen-8-ol (8.3) 1-Borneol (4.0) Isophorone (3.1) Camphene (1.3)	*A. niger*	Paper disc diffusion (10 μL/disc)	IZ diameter = 11 mm	
						Paper disc diffusion (0.15 to 5.0 μL/disc)	MIC = 5.0 μL/disc	
					*F. solani*	Paper disc diffusion (10 μL/disc)	IZ diameter = 9 mm	
						Paper disc diffusion (0.15 to 5.0 μL/disc)	MIC = 5.0 μL/disc	
Leaves	Aqueous extract	Maceration	Algeria	N.A.	*Fusarium* sp.	Paper disc diffusion (6.4 mg/disc in 100 µL)	No effect	[67]
						Agar dilution method (>0.62 mg/mL)	No effect	
	Petroleum ether					Paper disc diffusion (6.4 mg/disc in 100 µL)	No effect	
						Agar dilution method (>0.62 mg/mL)	No effect	
	Methanol					Paper disc diffusion (6.4 mg/disc in 100 µL)	IZ diameter = 17 mm	
						Agar dilution method	MIC = 0.08 mg/mL	
	Ethanol					Paper disc diffusion (6.4 mg/disc in 100 µL)	No effect	
						Agar dilution method (>0.62 mg/mL)	No effect	
	Ethyl acetate					Paper disc diffusion (6.4 mg/disc in 100 µL)	IZ diameter = 17.33 mm	
						Agar dilution method	MIC = 0.08 mg/mL	
	Chloroform					Paper disc diffusion (6.4 mg/disc in 100 µL)	No effect	
						Agar dilution method (>0.62 mg/mL)	No effect	

EOs: Essential Oils, MIC: Minimum Inhibitory Concentration, MFC: Minimum Fungicidal Concentration, IZ: Inhibition zone, N.A: Not available. Values are reported as in the original studies, with the original units and endpoints. Because the studies used different experimental methods, numerical values obtained with different endpoints or units are not directly comparable.

## 4. Mechanisms of Action of EOs and Extracts from *Artemisia* Species and Related Systems

Numerous alternative treatments have been explored to reduce reliance on synthetic chemical fungicides for food preservation. Plant extracts and EOs, which possess bioactive properties, may represent viable options for safeguarding stored products. Findings from various studies examining the efficacy of plant EOs against mycotoxigenic fungi suggest considerable variability in EO composition, which may contribute to differences in their antifungal activity. As mentioned previously, the chemical variability of EOs is attributed to varying ecological and geographical conditions as well as harvesting time [24,36,68].

The stage at which plants are harvested may affect the concentration of bioactive compounds in their EOs. For example, harvesting plants at specific growth stages can optimize the concentrations of phenolic compounds, terpenes, and other constituents with antifungal properties [69]. The extraction methodology of EOs also may also influence EO composition, and methods such as hydrodistillation or microwave-assisted distillation can yield oils with varying concentrations of bioactive compounds, thereby influencing their antifungal activity [58]. Additionally, drying methods used for plant parts can significantly impact the chemical composition of EOs. Traditional methods, such as shade drying, may preserve the chemical constituents of EOs. Al-Qudah et al. (2021) suggested that shade-dried samples may retain higher levels of EO contents than those dried using other techniques [70].

The antifungal activity of plant extracts primarily depends on the types and concentrations of bioactive compounds present. Different plants can contain various compounds such as phenolics, flavonoids, terpenoids, alkaloids, and saponins, each of which may have antifungal properties. These compounds may act synergistically or independently to inhibit fungal growth [71]. However, most studies do not focus on examining the chemical constituents of plant extracts. The antifungal potential of plant extracts also depends on the solvent used for extraction. Different solvents possess distinct chemical properties that influence the range of compounds they can extract from plant material. Consequently, solvent choice can produce extracts with varying antifungal activity. Polar solvents such as methanol are generally more effective in extracting a broader range of compounds due to their superior ability to penetrate plant tissues [72,73]. In contrast, non-polar solvents like petroleum ether may not extract as diverse a range of compounds, which can limit the antifungal efficacy of the resulting extract [74,75].

**Figure 2:**
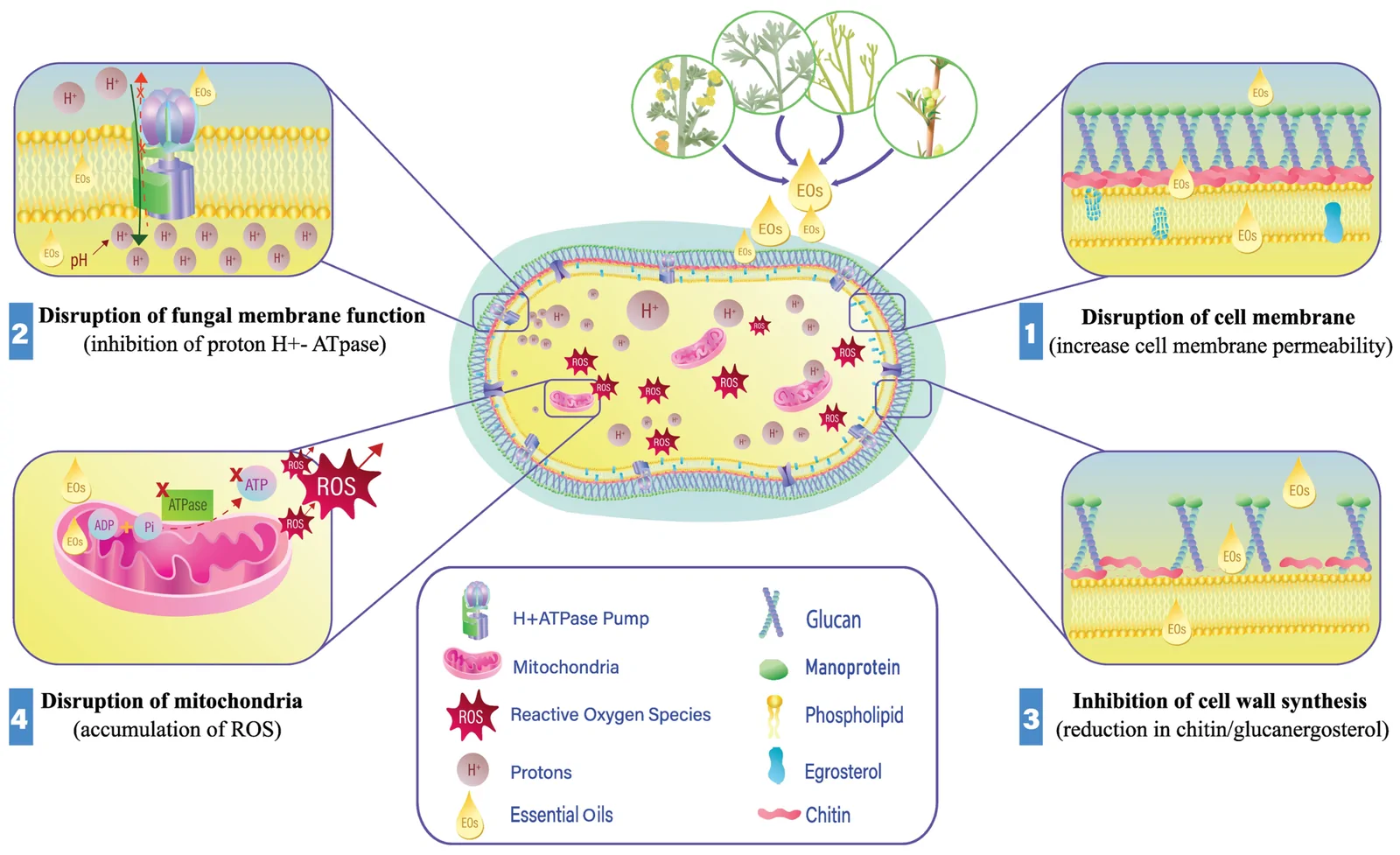
Schematic overview of the main antifungal mechanisms proposed for *Artemisia* essential oils: membrane disruption; cell wall interference; and mitochondrial dysfunction with ROS accumulation.

Despite extensive work on activity profiles, the precise mechanisms by which EOs and plant extracts exert antifungal effects remain only partially understood, and the available evidence has been generated at different levels. To make this distinction explicit, the mechanisms discussed below are presented in three categories: (i) mechanisms directly demonstrated for EOs or extracts of the four reviewed *Artemisia* species; (ii) mechanisms demonstrated for individual constituents identified in these *Artemisia* preparations; and (iii) mechanisms proposed on the basis of studies of other EOs, other plant extracts, or isolated compounds not reported in *Artemisia* species. Category (iii) evidence is retained as background to generate hypotheses for future work on *Artemisia* preparations and is explicitly identified as such; it should not be interpreted as direct evidence for the mode of action of *Artemisia* products. The major targets and processes proposed in the literature, which are summarized in Figure 2 and outlined below, span all three categories.

### 4.1. Inhibition of Cell Wall

The fungal cell wall surrounds the cell, protecting it from internal osmotic pressure, providing mechanical support, and maintaining cell shape [76]. It is a characteristic structure composed mainly of glucans, chitin and glycoproteins. Chitin, which is abundant in filamentous fungi, consists of β-1,4-linked N-acetylglucosamine and is covalently linked to β-1-3-glucan [77]. Disruption of this structure compromises cell integrity, leading to plasma membrane rupture and cell lysis [77]. EOs can interfere with cell wall components and weaken structural integrity, resulting in impaired growth and eventual cell death. For example, paeonol (2-hydroxy-4-methoxyacetone), a component commonly isolated from *Cynanchum paniculatum*, and not reported in the *Artemisia* preparations reviewed here, has been shown to alter the cell wall and reduce the content of both β-1,3-glucan and chitin in *Aspergillus flavus* [78]. Whether *Artemisia* EOs or extracts exert comparable effects on cell wall polysaccharides has not been directly demonstrated to date.

### 4.2. Disruption of Cell Membrane

EO components such as terpenes and phenolic compounds can interact with lipids in the fungal cell membrane. This interaction alters membrane permeability and fluidity, leading to leakage of ions, proteins, and other intracellular constituents and ultimately to cell death [79]. Ergosterol is a major component of the fungal membrane and acts as a bioregulator of fluidity, asymmetry and integrity [80]. Reductions in ergosterol content weaken membrane structure and function.

Several studies suggest that EOs can significantly reduce ergosterol levels, although none of these studies used *Artemisia* preparations. For instance, clove oil and its main compound, eugenol, cause a marked decrease in ergosterol content, compromising membrane integrity [81]. Tea tree oil has also been shown to disrupt the permeability barrier of cell membranes and to cause loss of chemiosmotic control [82]. These observations suggest that membrane disruption may constitute a central mechanism in EO-mediated antifungal activity and provide testable hypotheses for *Artemisia* EOs, whose main constituents are also lipophilic terpenoids. However, direct demonstration of membrane-level effects for *Artemisia* preparations is still lacking.

### 4.3. Disruption of Mitochondrial Function

Mitochondrial dysfunction may result from EO-mediated inhibition of mitochondrial ATPase activity, thereby decreasing ATP content and impairing mitochondrial dehydrogenase activity. Both effects may reduce ATP synthesis, ultimately depleting mitochondrial ATP to levels that may be insufficient to sustain normal cellular metabolic functions. These mitochondrial dysfunctions may, in turn, enhance ROS accumulation. For instance, the antifungal activity of EOs from dill (*Anethum graveolens* L.) results from their ability to disrupt the permeability barrier of the plasma membrane and from the mitochondrial dysfunction-induced ROS accumulation in *Aspergillus flavus* [83]. Some common components of EOs such as α-pinene and β-pinene have also been reported to destroy cellular integrity, inhibit respiration and ion transport processes and increase membrane permeability of mitochondria isolated from yeast [84]. Additionally, EOs may inhibit enzyme activities essential to fungal metabolism, thereby disrupting pathways required for survival [85]. They also induce oxidative stress by generating ROS, which damage cellular components and ultimately lead to apoptosis [86,87]. Among the constituents relevant to this review, thymol has been shown to exert fungicidal action against spores of *Aspergillus flavus* through ROS accumulation and induction of nitric oxide [86], and thymol has been identified as a minor constituent of *A. judaica* EOs [50].

### 4.4. Disruption of Fungal Membrane Function

The fungal plasma membrane H+-ATPase is an important enzyme that maintains the transmembrane electrochemical proton gradient necessary for nutrient uptake and regulate intracellular pH [88]. The inhibition of H+-ATPase can lead to intracellular acidification and cell death [89]. Isoeugenol and 4-methoxycinnamaldehyde, two EO components not reported in the *Artemisia* preparations reviewed here, have markedly inhibited growth of *Candida* isolates by the inhibition of H+-ATPase and membrane disruption [90]. In a study by Prakash et al. (2010), the minor components of the oil may have acted antagonistically, thereby reducing the activity of the major compound [91]. For instance, the use of *Piper betle* L. EOs, where eugenol was the major component (63.39%), demonstrated that eugenol was more effective as a fungal growth inhibitor than the EOs. Thus, it is difficult to correlate the activity of a complex mixture to a particular component and the same caution applies to *Artemisia* EOs, whose activity cannot be attributed to individual constituents without direct testing.

## 5. Effects of EOs and Extracts on Mycotoxin Production

Fungal growth inhibition is the most frequently reported outcome in studies of *Artemisia* products, but the ultimate goal of mycotoxin reduction or control depends on whether toxin biosynthesis itself is affected. In the studies reviewed here, growth inhibition and mycotoxin production were not always coupled, and both suppressive and stimulatory effects on toxin output have been reported.

### 5.1. Inhibition of Mycotoxin Production

Direct evidence for the suppression of mycotoxin biosynthesis by *Artemisia* EOs remains limited to a few studies. Leaf EOs of *A. herba-alba* completely inhibited mycelial growth of *P. aurantiogriseum* and *P. viridicatum* at concentrations of 0.89% (*v*/*v*) and 1.33% (*v*/*v*), respectively, and suppressed the production of the mycotoxins and secondary metabolites penicillic acid, terrestric acid, aurantiamine, brevianamide A, and xanthomegnin at a concentration of 0.44% (*v*/*v*) [45]. This study is notable because the toxin-inhibitory concentration was lower than the growth-inhibitory concentrations, indicating that *A. herba-alba* EOs can inhibit toxigenesis without fully arresting fungal growth. At the constituent level, piperitone, the dominant component of several *A. herba-alba* and *A. judaica* chemotypes, has been reported to suppress AFB1 production and aflatoxin biosynthetic gene expression in *Aspergillus flavus*, although this effect has not yet been validated using the complete *Artemisia* EOs from which piperitone is derived [56].

### 5.2. Stimulation of Mycotoxin Production at Sub-Inhibitory Doses: Evidence from Other Species

To our knowledge, no study has yet reported stimulation of mycotoxin production by *Artemisia* species preparations at sub-inhibitory concentrations. The evidence summarized in this section was obtained with EOs of other plant species to demonstrate that fungal growth and toxin production can respond independently to treatment concentration. Therefore, dose-dependent effects on mycotoxin production may not be inferred from growth-inhibition data alone. For instance, the production of the mycotoxin OTA by *Aspergillus carbonarius* after seven days of incubation was about 608.5 ± 34.4 ng/g on Czapek Yeast Agar (CYA) medium. The addition of 0.1% and 0.2% of *Salvia officinalis* EOs in the culture medium significantly stimulated OTA production with amounts equal to 1832.4 and 1916.4 ng/g CYA medium, respectively [92]. In another study, a concentration of 0.1 μL/mL of *Piper betle* L EOs stimulated production of AFB1 by *Aspergillus flavus*. However, the aflatoxin-inhibitory activity of the EOs improved with increasing concentrations, and at 0.6 μL/mL, it suppressed AF production by the mycotoxigenic isolate [91]. These results demonstrate that low doses of EOs may create stress conditions that can trigger the production of more secondary metabolites or mycotoxins as a possible defense mechanism by the fungi [91].

Taken together, these findings suggest that assuming any concentration of an EO to be safe for food applications may be unwarranted. Sub-inhibitory doses can trigger the opposite of the intended effect, whereas adequate doses can suppress toxin production. Although the stimulatory observations in this review involve non-*Artemisia* EOs (*S. officinalis* and *P. betle*), they justify evaluating *Artemisia* EOs and extracts not only for growth inhibition but also for dose-dependent effects on mycotoxin production before their use as biocontrol agents.

## 6. Safety Assessment of EOs and Extracts from *Artemisia* Species

Safety assessment of EOs is important as they are widely used in food, cosmetics and medicinal products. Despite being derived from natural sources, some constituents may exert toxic risks, particularly at high doses or upon prolonged exposure. Available data for the four species considered in this review indicate a mixture of compound-specific risks and relatively wide safety margins in cell and animal models, as summarized below.

### 6.1. Safety Assessment of EOs and Extracts from Artemisia absinthium

Safety concerns for consideration regarding *A. absinthium* mainly relate to thujone, a neuroactive monoterpene. Long-term administration of *A. absinthium* preparations has been associated with neurotoxic effects that may be attributable to thujone [93], and multiple in vivo toxicity studies have shown dose-dependent adverse outcomes for this compound [93]. Rivera et al. (2014) reported that intracerebroventricular administration of α-thujone suppressed stress-induced recruitment of GABAA receptors, which may reflect blockade of the benzodiazepine binding site or another locus within the GABAA complex, providing mechanistic support for its neurotoxicity [94].

On the other hand, whole EOs and extract studies suggest relatively limited cytotoxicity within certain dose ranges. The antiproliferative effects of *A. absinthium* EOs were evaluated against normal human skin fibroblasts (HSFs) and several cancer cell lines (A431, MCF-7, PANC-1) using the sulforhodamine B assay, and the EOs showed only limited cytotoxic effects against all tested lines [95]. Taherkhani (2014) reported that the IC50 of *A. absinthium* EOs was lower for human tumour cells than for healthy human cells, indicating preferential activity against cancer cells and suggesting relatively low adverse effects for normal cells at active concentrations [96]. In acute oral toxicity testing, methanolic extracts of *A. absinthium* administered to mice up to 5000 mg/kg produced no observable toxicity [97]. Overall, current data suggest that *A. absinthium* extracts and EOs can be used at doses that are cytocompatible for several cell types and non-toxic in acute oral models, but that thujone-driven neurotoxicity remains a key concern, particularly for long-term or high-dose exposure.

### 6.2. Safety Assessment of EOs and Extracts from Artemisia campestris

Data on the safety of *A. campestris* EOs are scarce and warrant caution. Judzentiene et al. (2010) reported that *A. campestris* EOs dominated by caryophyllene oxide were notably toxic, suggesting that specific chemotypes can display significant toxicity [98]. The absence of broad in vivo or detailed mechanistic studies makes it difficult to quantify risk, but this report points to the importance of chemotype screening and careful dose selection when considering *A. campestris* EOs.

### 6.3. Safety Assessment of EOs and Extracts from Artemisia herba-alba

An ethanolic extract of *A. herba-alba* was associated with genotoxic effects in mice and reduced the viability of cultured mouse bone marrow cells. In vivo, it lowered the mitotic index and raised the frequency of sister chromatid exchanges and micronuclei in bone marrow and peripheral blood cells, with statistically significant effects at 375 and 500 µg/mL [99]. In cultured bone marrow cells, viability was significantly reduced at the same two concentrations, with IC50 values of 484.38, 513.33, and 490.39 µg/mL after 24, 48, and 72 h of exposure. On the other hand, EOs from *A. herba-alba* showed no significant cytotoxicity in macrophages (RAW 264.7), microglia, or keratinocytes at concentrations up to 0.32 µL/mL in the MTT assays [49]. Acute oral toxicity has been examined in three single-dose studies, which report differing results. In a study conducted according to the OECD guideline 423, oral administration to female Wistar rats caused no mortality at 300 mg/kg but mortality at 2000 mg/kg, yielding an estimated LD50 of 1000 mg/kg and suggesting a classification as weakly toxic [100]. In another study in rats, mortality occurred at 2000 mg/kg and the LD50 was estimated between 50 and 500 mg/kg, corresponding to moderately toxic on the Hodge and Sterner scale [101], while oral administration to mice at doses of 0.5–2 g/kg caused no mortality or adverse effects during two weeks of observation, apart from transient restlessness at the highest dose [102]. One further acute toxicity study in mice, using intraperitoneal administration, reported an LD50 of 615 mg/kg, corresponding to slightly toxic on the Hodge and Sterner scale [103].

Taken together, genotoxic, cytotoxic, and lethal effects were observed mainly at high doses, and the acute toxicity results are heterogeneous across studies. Because the available data derive from single-dose exposures, the safety of *A. herba-alba* EOs for food applications has not yet been established, as discussed in the general considerations above.

### 6.4. Safety Assessment of EOs and Extracts from Artemisia judaica

Safety information for *A. judaica* is more limited but suggests a concentration-dependent window of tolerability. Abu-Darwish et al. (2016) assessed the cytotoxicity of *A. judaica* EOs in macrophages (RAW 264.7) and HepG2 hepatocellular carcinoma cells using MTT assays and found no significant cytotoxicity up to 0.32 µL/mL in either cell type [65]. In a more detailed Alamar Blue assay, *A. judaica* oil exhibited concentration-dependent effects on RAW 264.7 macrophages: exposure to concentrations up to 20 µg/mL did not significantly reduce cell viability, whereas higher concentrations became cytotoxic [104]. These results indicate that *A. judaica* EOs are well tolerated within a defined concentration range in mammalian cells, but that increasing doses beyond this window can compromise cell viability, highlighting the need to respect upper limits when designing applications.

## 7. Conclusions

*Artemisia* species, including *A. absinthium*, *A. campestris*, *A. herba-alba*, and *A. judaica*, show potential as natural antifungal agents for controlling mycotoxigenic fungi. The reviewed studies provide reasonable evidence that their EOs and extracts inhibit the growth of these fungi under in vitro conditions and exhibit additional antioxidant and antimicrobial activities, which has led to their proposal as candidates to reduce reliance on synthetic chemical preservatives. However, most of the data comes from laboratory assays based on growth endpoints, and none of the retrieved studies tested the preparations in real food systems, such as stored commodities or processed products. Direct evidence on mycotoxin production is even scarcer since only one study measured toxin levels in fungal cultures, using a qualitative method, and growth inhibition cannot be assumed to translate into suppression of toxin synthesis.

Mechanistic understanding is similarly incomplete and direct experiments with *Artemisia* preparations are rare. Mechanistic events occurring when fungi are exposed to one of the *Artemisia* species EOs, with their complex and variable mixtures of mono- and sesquiterpenes, remain unresolved. Compositional variability may itself constitute a practical problem, because the reviewed EOs differ markedly in their major components and many studies did not report complete chemical profiles, which may complicate both the interpretation of results and any future standardization.

Before food applications can be considered, their activity would need to be confirmed in real food matrices and under realistic storage conditions; the relationship between concentration and toxin production needs to be characterized, including the possibility that sub-inhibitory doses might stimulate mycotoxin synthesis, as reported for some other essential-oil systems; reproducible preparations with defined composition would likely be required; and safety would need to be evaluated on the basis of oral or dietary exposure, since current toxicity data rest largely on acute single-dose studies. Whether the promising in vitro activity of *Artemisia* preparations can be translated into safe and effective use in the food chain may depend on these investigations.

## Data Availability

No new data were created or analyzed in this study. Data sharing is not applicable to this article.
